# Iron modulation drives biofilm formation and virulence enzyme production in emerging clinical *Candida* species: implications for diagnostics and therapeutics

**DOI:** 10.3389/ffunb.2025.1746357

**Published:** 2026-01-21

**Authors:** Shabnam Kumari, Zinnu Rain, Pradyot Prakash, Deepak Kumar, Munesh Kumar Gupta, Ashish Kumar Singh, Ragini Tilak

**Affiliations:** 1 Department of Microbiology, Institute of Medical Sciences, Banaras Hindu University, Varanasi, India; 2 Department of Biochemistry, Patna University, Patna, India

**Keywords:** biofilm-associated infections, clinical candidemia, echinocandin resistance, emerging fungal pathogens, fungal virulence, iron homeostasis

## Abstract

**Background:**

The changing epidemiology of candidemia indicates a rise in non-albicans *Candida* species, especially resistant *Candida auris* and emerging *Candida utilis*. Although iron impacts fungal virulence, its role in these species remains poorly understood. This study investigates how manipulating iron levels influences biofilm formation, virulence enzymes, and antifungal susceptibility in clinical isolates.

**Methods:**

A total of 216 isolates of *Candida utilis*, *Candida albicans*, and *Candida auris* from bloodstream infections over two years were identified via phenotypic methods, MALDI-TOF MS, VITEK 2, and 18S rRNA PCR. Susceptibility was tested using disc diffusion and broth microdilution with ferrous sulphate (FeSO_4_). Virulence enzyme activities and biofilm formation were assessed under iron-rich and control conditions.

**Results:**

*Candida auris* showed multidrug resistance, especially to fluconazole and caspofungin, with iron increasing caspofungin MICs up to 16-fold. *Candida utilis* exhibited strong biofilm formation and increased phospholipase and proteinase activities in the presence of FeSO_4_, and also showed 4- to 32-fold increases in fluconazole resistance. Biofilm biomass was unaffected by iron, but enzyme activities varied by species and enzyme. *Candida albicans* had high proteinase and haemolysin activity but responded minimally to iron.

**Conclusions:**

Iron differentially influences virulence−associated traits (biofilm−related enzyme activities) and antifungal resistance across these *Candida* species. *C. utilis* exhibits iron−responsive increases in phospholipase and proteinase activities together with amplified azole resistance, while *C. auris* shows iron−linked enhancement of echinocandin resistance and sustained expression of key virulence−associated enzymes. These results underscore the importance of accounting for host iron levels and species-specific responses when managing candidemia and indicate the potential for therapies targeting iron.

## 1 Introduction

The global epidemiology of candidemia has undergone significant changes in recent years, with the prevalence of non-albicans *Candida* (NAC) species rising, overtaking *Candida albicans* as the leading cause of bloodstream fungal infections. Although *Candida glabrata* (*Nakaseomyces glabratus*) is common in developed regions, species such as *Candida tropicalis* and *Candida parapsilosis* are frequent in resource-limited settings. The emergence of *Candida auris*, a multidrug-resistant and rapidly spreading pathogen, has heightened the clinical challenge by increasing antifungal resistance and complicating species identification (Mulet Bayona et al., 2023; Pyrpasopoulou et al., 2025). During this shift, *Candida utilis*, formerly valued for its benefits to industrial biotechnology and food processing because of its recognised safety and metabolic flexibility, is now emerging as a clinically relevant fungal pathogen (Carranza-Méndez et al., 2022). Recent surveillance shows its remarkable ability to adapt to hospital environments, including changes in pH, temperature, and osmolarity, and to resist antifungal treatments, as well as a concerning tendency to form biofilms on indwelling medical devices (Scoppettuolo et al., 2014; Singh et al., 2024). Importantly, while the emergence of *C. auris* is often linked to antibiotic resistance and hospital outbreaks, the clinical resurgence of *C. utilis* seems to be driven by a combination of traditional virulence traits, such as biofilm formation and enzyme production, and notable ecological adaptability. *C. utilis* is increasingly associated with severe infections, including candidemia and device-related mycoses, emphasising the need for careful species identification and infection management strategies to address the evolving spectrum of *Candida* pathogens (Vila et al., 2020; Eix and Nett, 2025). Candidiasis is still a significant “disease of the diseased”, affecting those who have substantial underlying morbidity and compromised immunity (Patel, 2022). Fungi previously deemed harmless are increasingly acknowledged for their ability to cause considerable morbidity and mortality in both immunocompetent and especially immunocompromised individuals. This escalating public health issue underscores the need for thorough species identification and accurate antifungal susceptibility testing to guide effective management in both routine and emergent clinical settings (Lass-Florl et al., 2024).

Key to *Candida* pathogenicity is the utilisation of a myriad of virulence factors, including efficient host tissue adhesion, adaptive stress response mechanisms, secretion of hydrolytic enzymes, and biofilm formation (Farhan et al., 2024). Secreted enzymes, including proteinases, phospholipases, and haemolysins, enable the invasion and manipulation of host tissues, circumvent immunological defences, and directly cause tissue damage. The breakdown of host cell membranes by phospholipase facilitates rapid fungal dissemination, while extracellular haemolysin provides a survival benefit by sequestering iron from the host. The interaction of these attributes, therefore, governs the shift from commensalism to aggressive pathogenicity (Sahoo and Rao, 2024).

Recent advancements highlight the crucial role of iron homeostasis in *Candida* infection by regulating virulence factors that significantly impact fungal proliferation, biofilm development, maturation, and antifungal resistance. Iron homeostasis is crucial for fungal pathogenesis, serving as a nutrient and environmental signal that influences virulence and antifungal tolerance in *Candida* species. Iron supports key metabolic pathways, such as respiration, DNA synthesis, and enzyme function, and is regulated by Sfu1 and Gsf2/Cre1. Under iron-rich conditions, *Candida* upregulates transporters like Ftr1 and siderophore-independent systems, promoting growth and hyphal development. Iron scarcity triggers Hog1-MAPK and EFG1 pathways, enhancing adhesion and biofilm formation, which are essential for tissue invasion and device infections. Biofilm development is iron-sensitive excess iron boosts extracellular matrix via cell wall integrity signalling and Mkc1, while deficiency leads to dispersed, invasive biofilms through Gcn4-dependent amino acid starvation responses. In *C. albicans*, iron limitation affects drug susceptibility, but supplementation restores matrix and tolerance. In *C. auris*, iron influences biofilm gene expression, linking nutrient status to persistence. Iron modulates antifungal resistance by stabilising ergosterol (reducing azole efficacy) and buffering echinocandin activity through chitin synthesis. Iron-driven ROS and efflux pumps confer multidrug tolerance, reversible with chelators. These pathways show that iron reprograms *Candida* from commensalism to pathogenicity, especially in iron-variable niches such as blood or devices. Our study extends this framework by quantifying iron’s species-specific effects on enzyme activities and MICs in clinical isolates *of C. utilis, C. albicans*, and *C. auris* to improve the diagnosis and treatment of candidemia.

## 2 Materials and methods

### 2.1 Strain collection and identification

This study was approved by the Ethical Committee of the Institute, Institute of Medical Sciences, BHU, Varanasi, under the reference number IMS/IEC/2024/7332. A total of 216 clinical isolates of *Candida* species, including *Candida utilis*, *Candida albicans*, and *Candida auris*, were collected over two years from August 2022 to July 2024. These were isolated from clinical blood specimens obtained from patients at Sir Sunder Lal Hospital, Banaras Hindu University, Varanasi.

Initial identification of isolates was performed using standardised phenotypic methods. Cultures were grown on Sabouraud’s dextrose agar (SDA) and incubated aerobically at 37°C for 24 hours to ensure viability and purity. Colony morphology and pigmentation were examined on Cornmeal agar and HiChrome *Candida* agar, respectively, facilitating presumptive species differentiation based on colouration patterns (Baradkar et al., 2010). Microscopic examination included Gram’s staining and analysis of germ tube formation to differentiate *C. albicans* from NAC species initially. Cornmeal agar supplemented with Tween 80 was utilised to assess chlamydospore production and esterase activity, a phenotypic marker relevant for species identification (Bottcher et al., 2016). Definitive species identification was confirmed using Vitek 2 and advanced MALDI-TOF MS (Bruker Daltonik GmbH, Bremen, Germany) at PGIMER, Chandigarh, India.

To complement phenotypic methods, molecular identification was conducted through conventional polymerase chain reaction (PCR) targeting the 18S rRNA gene, using primers (Forward 5-ACTCAACACGGGGAAACT-3 and Reverse 3-ATTCCTCGTTGAAGAGCA-3) that were previously validated by Embong et al. (2008) (Embong et al., 2008). The primer pair amplified a 401 bp fragment, demonstrating high specificity for confirming *Candida*. PCR products were visualised via gel electrophoresis to verify the amplicon size and clarity. Reference strains *Candida albicans* ATCC 90028 and *Candida auris* CDC B11903 were used as controls during identification and subsequent assays. These strains were stored at −80°C in Brain Heart Infusion broth supplemented with 20% glycerol (Merck, Darmstadt, Germany). All clinical isolates were maintained as stock cultures in 15% glycerol broth at −20°C and revived on SDA before each experimental procedure to ensure consistency and reproducibility.

#### 2.1.1 Identification employing Vitek 2 test

A sterile swab was used to collect an adequate number of fungal colonies from a pure culture, which were suspended in 3.0 mL of sterile saline (0.5% NaCl) in a clear plastic test tube. The turbidity was adjusted to a range of 1.80-2.0 using the DensiChekTM system. Identification cards were inoculated with the yeast suspension using a vacuum apparatus. The test suspension tube was placed into a cassette, with the transfer tube inserted. The loaded cassette was then placed in a vacuum chamber, allowing the yeast suspension to flow into the microchannels and fill all test wells. After 18 hours of incubation, the results on the card were compared to an identification database to determine the unknown organism (Melhem et al., 2013).

#### 2.1.2 Identification employing MALDI TOF MS

*Candida* isolates were identified using MALDI-TOF mass spectrometry at PGIMER, Chandigarh, with the on-plate formic acid extraction method (Ghosh et al., 2015). Briefly, 2–3 fresh *Candida* colonies were collected with a loop, transferred to a microcentrifuge tube containing 1 mL of sterile double-distilled water, and vigorously vortexed. The suspension was centrifuged at 13,000 rpm for 2 minutes, and the pellet was collected. This process was repeated twice, with the final pellet resuspended in 50 µL of sterile double-distilled water. One microlitre of the suspension was spotted onto the MALDI target plate and air-dried. Next, 0.5 µL of 98% formic acid was applied to the spot and allowed to air-dry. Subsequently, 0.8 µL of matrix solution (comprising 50 µL acetonitrile, 2.5 µL trifluoroacetic acid, 1 mg α-cyano-4-hydroxycinnamic acid, and 47.5 µL sterile double-distilled water) was added to each spot and air-dried. MALDI-TOF MS analysis was performed using a MALDI Microflex LT mass spectrometer (Bruker Daltonik GmbH, Bremen, Germany). Identification scores equal to or greater than 2.0 were considered reliable for species-level assignment, while scores between 1.7 and 1.99 indicated genus-level identification. Scores below 1.7 were deemed inconclusive and excluded from further analysis.

### 2.2 Antifungal susceptibility testing by the disc diffusion method

The colonies were suspended in 5 mL of 0.85% sterile normal saline, and the turbidity was adjusted to match a 0.5 McFarland standard. For susceptibility testing, Mueller-Hinton agar supplemented with 2% glucose and 0.5 µg/mL methylene blue was used. A sterile swab was employed to inoculate the agar surface, creating a lawn culture by rotating the plate 180° and streaking in three directions. Antifungal discs of fluconazole (25 µg), itraconazole (10 µg), amphotericin B (100 U), voriconazole (10 µg), and caspofungin (10 µg) were carefully placed on the inoculated plates. The plates were incubated at 37°C for 18–24 hours, and the zone diameters were measured and interpreted according to CLSI guidelines.

### 2.3 Determination of antifungal activity by microdilution method in the presence of ferrous sulphate

The antifungal susceptibility of *Candida* isolates was carefully studied in a controlled laboratory environment, following the CLSI M27-A3 guidelines. The antifungal agents tested included fluconazole, isovuconazole, and caspofungin, supplemented with iron sulphate at concentrations of 0, 20, 40, 80, and 160 μg/mL. Serial dilutions of these agents were prepared using RPMI-1640 medium and dispensed into a 96-well round-bottom microtiter plate, with each well containing 100 μL of the diluted drugs. Harvested cells were suspended in RPMI-1640 medium to approximately 5.0 × 10² to 2.5 × 10³ cells/mL, and 100 μL of this inoculum was added to each well, resulting in a total volume of 200 μL per well. After a 24-hour incubation at 35 °C, the susceptibility patterns of the isolates were analysed according to the CLSI M27-A3 guidelines. MIC endpoints were visually determined as the lowest drug concentration that inhibited 50% of visible growth relative to the drug-free control wells (for azoles and echinocandins). Quality control strains (*Candida parapsilosis* ATCC 22019 and *Candida krusei* ATCC 6258) were included in each run to ensure the accuracy and reproducibility of the testing process. These susceptibility profiles were thoroughly evaluated to identify intrinsic and emerging resistance patterns among *Candida utilis*, *Candida albicans*, and *Candida auris* isolates.

### 2.4 Determination of phospholipase activity in the presence of ferrous sulphate

Phospholipase activity was assessed on egg yolk agar plates supplemented with FeSO_4_ (0, 20, 40, 80, 160 µg/mL) to mimic host iron availability, as previously described (Chen et al., 1997). Egg yolk agar (8% v/v) was prepared with the specified FeSO_4_ concentrations; control plates (0 µg/mL) served as baseline references. A 5 µL drop of 2 McFarland-standardised *Candida* suspension was spotted in triplicate and incubated aerobically at 37°C for 5 days.

Activity was quantified by measuring precipitation zone diameters (egg yolk opacity). The Pz index was calculated as: colony diameter/(colony + precipitation zone diameter). Categories: negative (Pz=1.0), very low (0.90-0.99), low (0.80-0.89), high (0.70-0.79), very high (Pz ≤ 0.69). *C. albicans* ATCC 90028 served as the positive control. Assays were conducted in triplicate; means ± SD were calculated.

### 2.5 Determination of proteinase activity in the presence of ferrous sulphate

Proteinase activity assay measures proteolytic enzyme production, which plays a vital role in fungal virulence by promoting tissue invasion and immune evasion. This was evaluated using the albumin agar plate method, as described earlier (Sacristan et al., 2011). Briefly, BSA agar plates were prepared by supplementing standard agar medium with 1% (w/v) bovine serum albumin as the sole protein substrate and adding graded concentrations of FeSO_4_ (0, 20, 40, 80, and 160 μg/mL), adjusted to a pH of 5.0, to mimic iron-rich conditions. Control plates without FeSO_4_ were prepared alongside to serve as baseline references. *Candida* isolates were inoculated onto the surface of these plates using spot inoculation (6 mm) and incubated aerobically at 37°C for five days. During incubation, secreted proteinases degrade the albumin in the surrounding medium, resulting in clear zones of proteolysis around the colonies. After incubation, the diameter of these proteolytic zones was measured and expressed relative to the colony diameter to calculate the Proteinase Activity Index (Prz), which is the ratio of the colony diameter to the total diameter of the colony plus the proteolytic zone. A lower Prz value indicates higher proteinase activity. Triplicate measurements were performed to ensure reliability, and mean values with standard deviations were computed.

### 2.6 Determination of esterase activity in the presence of ferrous sulphate

Esterase activity plays a key role in the pathogenicity of *Candida*, as esterases aid in lipid metabolism and invasion of host tissues. It was evaluated in clinical *Candida* isolates using the Tween 80 opacity test, as described by Slifkin (2000) (Slifkin, 2000). In brief, the assay medium comprised a modified basal agar supplemented with 1% (v/v) Tween 80 and 0.01% (w/v) calcium chloride, which helps precipitate calcium salts of fatty acids released by esterase activity. To examine the effect of iron, ferrous sulphate (FeSO_4_) was added into the medium at specific concentrations (0, 20, 40, 80, and 160 μg/mL) to simulate iron-rich conditions.

A total of ten microliters of *Candida* isolates (2 McFarland’s turbidity) was spot-inoculated onto the surface of the Tween 80-containing agar plates, both with and without FeSO_4_ supplementation, and incubated aerobically at 30°C and examined daily for up to 10 days. During incubation, esterase enzymes hydrolyse Tween 80, releasing oleic acid, which, in combination with calcium ions, forms insoluble precipitates around the colonies. The colony diameter (a) and the total diameter of the colony plus the precipitation zone (b) were measured. Esterase activity was quantified as the Ez value (a/b), as outlined by Price et al. (1982). All assays were performed in triplicate to ensure consistent, reproducible results.

### 2.7 Determination of haemolysin activity in the presence of ferrous sulphate

Haemolysins facilitate fungal invasion by lysing host erythrocytes and releasing iron, an essential nutrient for growth and pathogenicity. The haemolysin activity of clinical *Candida* isolates was assessed using a modified blood agar assay, as described earlier (Luo et al., 2001). Briefly, blood agar plates were prepared by supplementing Sabouraud’s dextrose agar with 3% glucose and 7% sterile fresh sheep blood, along with varying concentrations of FeSO_4_ (0, 20, 40, 80, and 160 μg/mL) to simulate iron-rich microenvironments similar to host tissues. Control plates without FeSO_4_ were used to establish a baseline haemolytic activity.

A total of 10 microliters of *Candida* isolates (2 McFarland’s turbidity) were spot inoculated onto the blood agar surface and incubated aerobically at 30°C, monitored daily for 3 days. Haemolysin activity was detected by observing clear or greenish halos (zones of haemolysis) surrounding *Candida* colonies, indicative of erythrocyte lysis. Zones were measured in millimetres to quantify haemolytic activity. The control strain used was *Candida albicans* ATCC 90028. The Haemolytic Index (Hz) was calculated as the ratio of the diameter of the colony to the diameter of the colony plus the zone of haemolysis. A lower Hz value indicates higher haemolysin activity, as reflected by a comparatively larger lytic zone. Experiments were conducted in triplicate to ensure reproducibility and accuracy of results.

### 2.8 Impact of ferrous sulphate on biofilm-forming capacity of *Candida* species

Biofilm formation is a vital virulence trait, allowing fungal cells to adhere to both living and non-living surfaces, resist antifungal treatments, and evade host immune responses. Therefore, the effect of ferrous sulphate (FeSO_4_) on the biofilm-forming capacity of clinically isolated *Candida* species was examined using a standardised microtiter plate assay as described by Singh et al., with minor modifications, to assess biofilm formation at different iron levels (Singh et al., 2017).

Clinical isolates of *Candida utilis*, *Candida albicans*, and *Candida auris* were initially cultured on Sabouraud’s dextrose agar to confirm viability and purity. They were then inoculated into Brain Heart Infusion (BHI) broth at 37°C and diluted 1:100. Standardised inocula were prepared by adjusting the suspension of the clinical isolates to approximately 1×10^6^ cells/mL. A 100 μL aliquot of each *Candida* suspension was added to sterile 96-well flat-bottom microtiter plates containing 100 μL of BHI broth with incremental concentrations of FeSO_4_ (0, 4, 8, 32, 64, and 128 μg/mL). To mimic iron-enriched microenvironments, the specified concentrations were doubled to achieve the desired levels.

The plates were incubated at 37°C without agitation for 48 hours to allow biofilm development. After incubation, non-adherent cells were gently removed by washing the wells with sterile phosphate-buffered saline (PBS), and the attached biofilm biomass was quantified using crystal violet staining. In brief, *Candida* cells adhered, heat-fixed at 60°C, stained with 0.5% crystal violet, washed to remove excess stain, and air-dried. The dye was then dissolved using an ethanol-acetone mixture (80:20 v/v). Absorbance was recorded at 570 nm with an ELISA reader (MultiSkan SkyHigh, Thermo Scientific). This optical density directly reflects the amount of biofilm produced.

## 3 Results

### 3.1 Identification of clinical isolates

A total of 216 clinical isolates of *Candida* species, including *Candida utilis*, *Candida albicans*, and *Candida auris*, were recovered from diverse clinical samples collected at Sir Sunder Lal Hospital, Banaras Hindu University, over two years from August 2022 to August 2024. Preliminary identification was based on phenotypic features, colony morphology, and colour differentiation on selective media, followed by confirmatory identification through biochemical assays, MALDI-TOF MS, VITEK 2, and PCR targeting the 18S rRNA gene.

**Figure 1:**
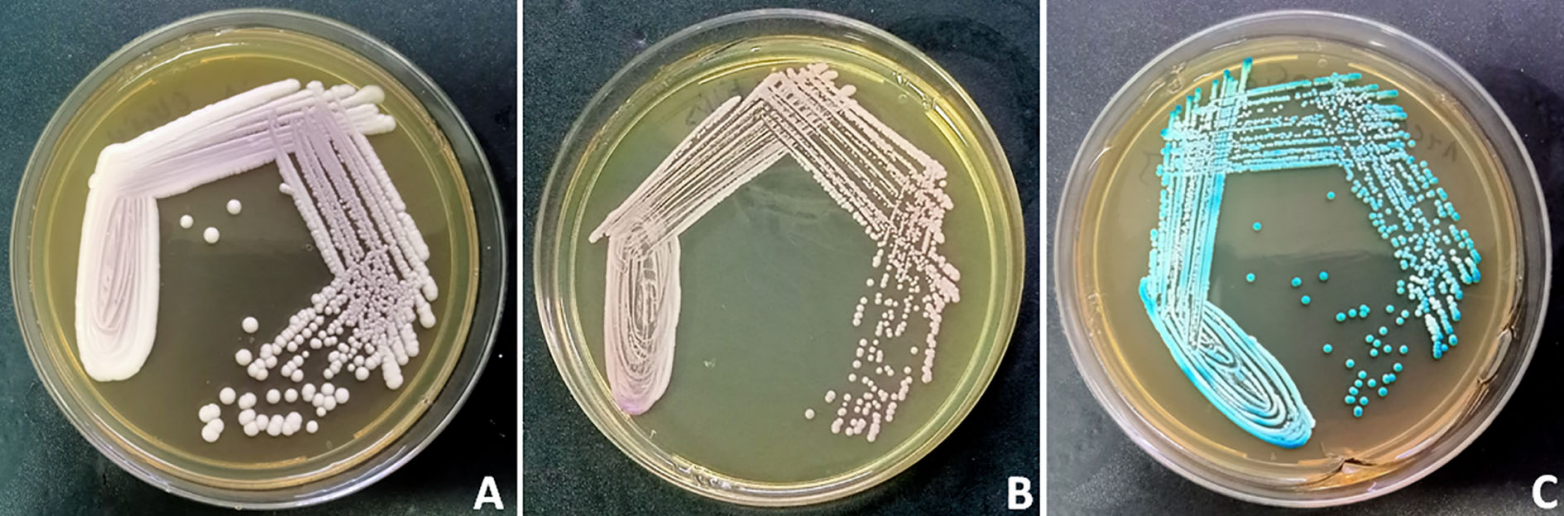
The attached image displays representative streak plates of three clinically significant *Candida* species on HiChrome *Candida* agar, illustrating their distinctive colony morphologies and pigmentation as part of the species identification process. **(A)** shows *Candida auris* as pinkish to cream, opaque colonies; **(B)** represents *Candida utilis* with light pink colonies; and **(C)** demonstrates *Candida albicans* forming green, smooth colonies. Accurate visual differentiation on chromogenic media facilitates prompt species-level identification, critical for guiding diagnostics and therapeutic decisions in candidemia and related infections.

On HiChrome *Candida* agar, *C. auris* formed distinct pinkish-cream, opaque colonies (**Figure 1A**), while *C. utilis* produced light pink colonies (**Figure 1B**). In contrast, *C. albicans* exhibited green, smooth colonies (**Figure 1C**), facilitating initial differentiation.

**Figure 2:**
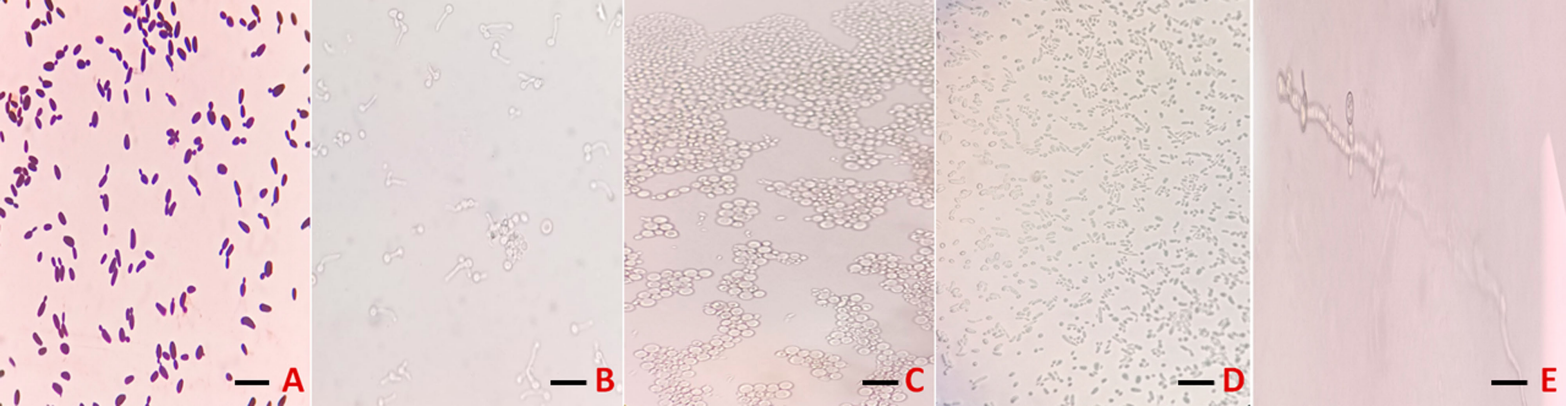
Microscopic differentiation of clinical *Candida* species (scale bar = 5 µm). **(A)** Gram staining (yeast morphology) **(B)***C. albicans* germ tube formation in serum **(C)** Larger ovoid *C. auris* cells on cornmeal agar **(D)** Smaller ovoid *C. utilis* cells on cornmeal agar **(E)** Terminal chlamydospore formation confirming *C. albicans*.

Likewise, microscopic analysis, including Gram staining (**Figure 2A**), germ tube formation (**Figure 2B**), and chlamydospore assessment (**Figure 2C-E**), further supported species-level identification and distinguished morphotypes. *C. albicans* exhibited germ tube formation (**Figure 2B**) and chlamydospore production (**Figure 2E**); *C. utilis* and *C. auris* showed budding yeast cells without these features, with *C. auris* exhibiting a bigger ovoid yeast-like appearance compared to *C. utilis* (**Figure 2C, D**).

**Figure 3:**
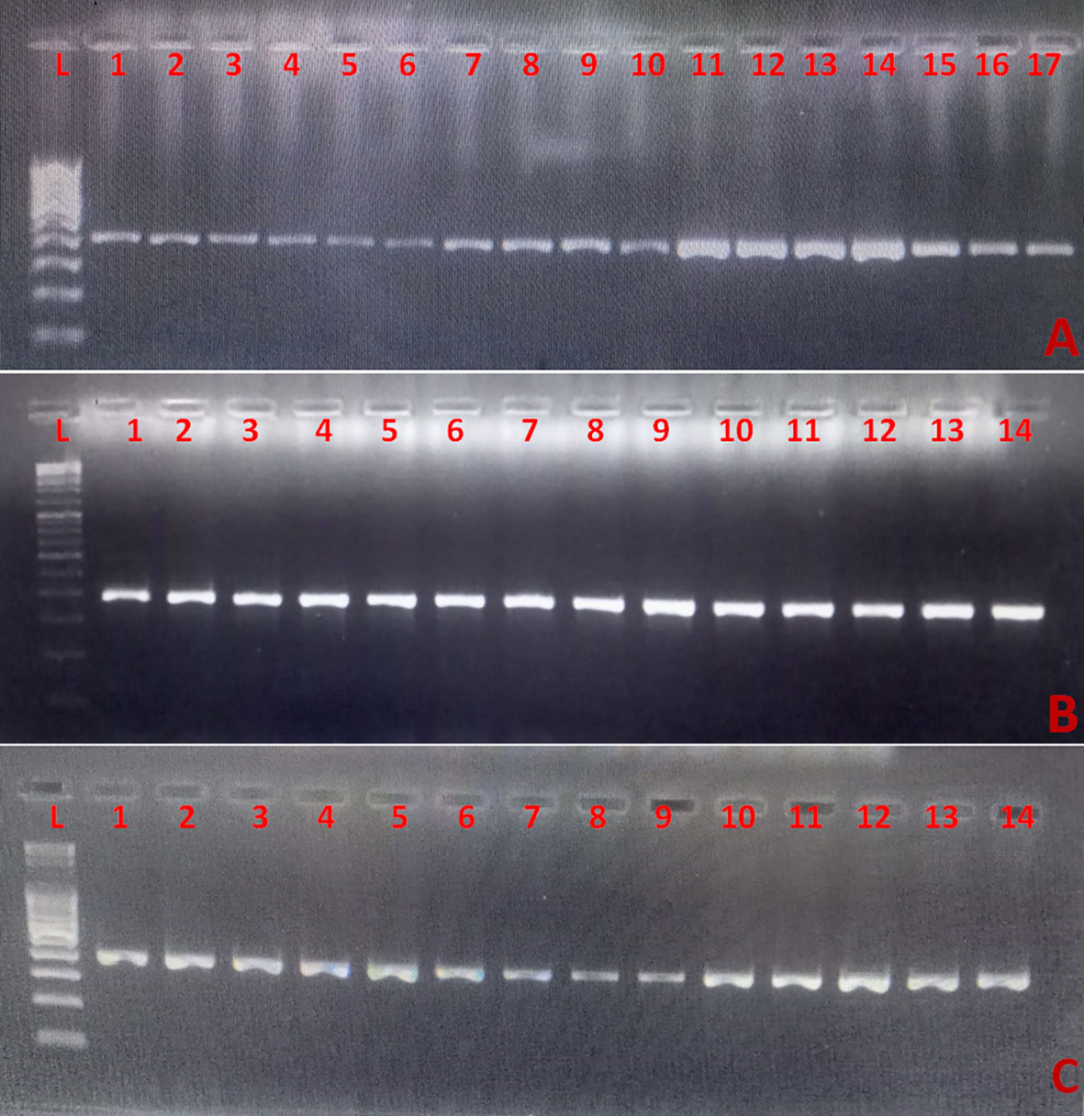
Agarose gel electrophoresis of PCR products from the 18S rRNA gene confirms species-level validation of clinical *Candida* isolates, cross-verified with MALDI-TOF/VITEK. Panels **(A)** (*C. utilis*, lanes 1-17), **(B)** (*C. albicans*, lanes 1-14), and **(C)** (*C. auris*, lanes 1-14) show consistent 401 bp amplicons. Lanes labelled “L”: 100 bp DNA ladder. Positive controls: *C. albicans* ATCC 90028 and *C. auris* CDC B11903 (run in parallel, data not shown).

Additionally, definitive confirmation was achieved through PCR amplification of the 18S rRNA gene. The three panels demonstrate species-level validation for multiple isolates of *Candida utilis*, *Candida albicans*, and *Candida auris*, respectively (**Figure 3A-C**). Lane patterns show consistent amplicon sizes (401 bp) across representative isolates, confirming molecular accuracy. Furthermore, MALDI-TOF MS and VITEK 2 provided definitive species identification, with scores ≥2.0 confirming each isolate. 

### 3.2 Antifungal susceptibility profile by the disc diffusion method

**Figure 4:**
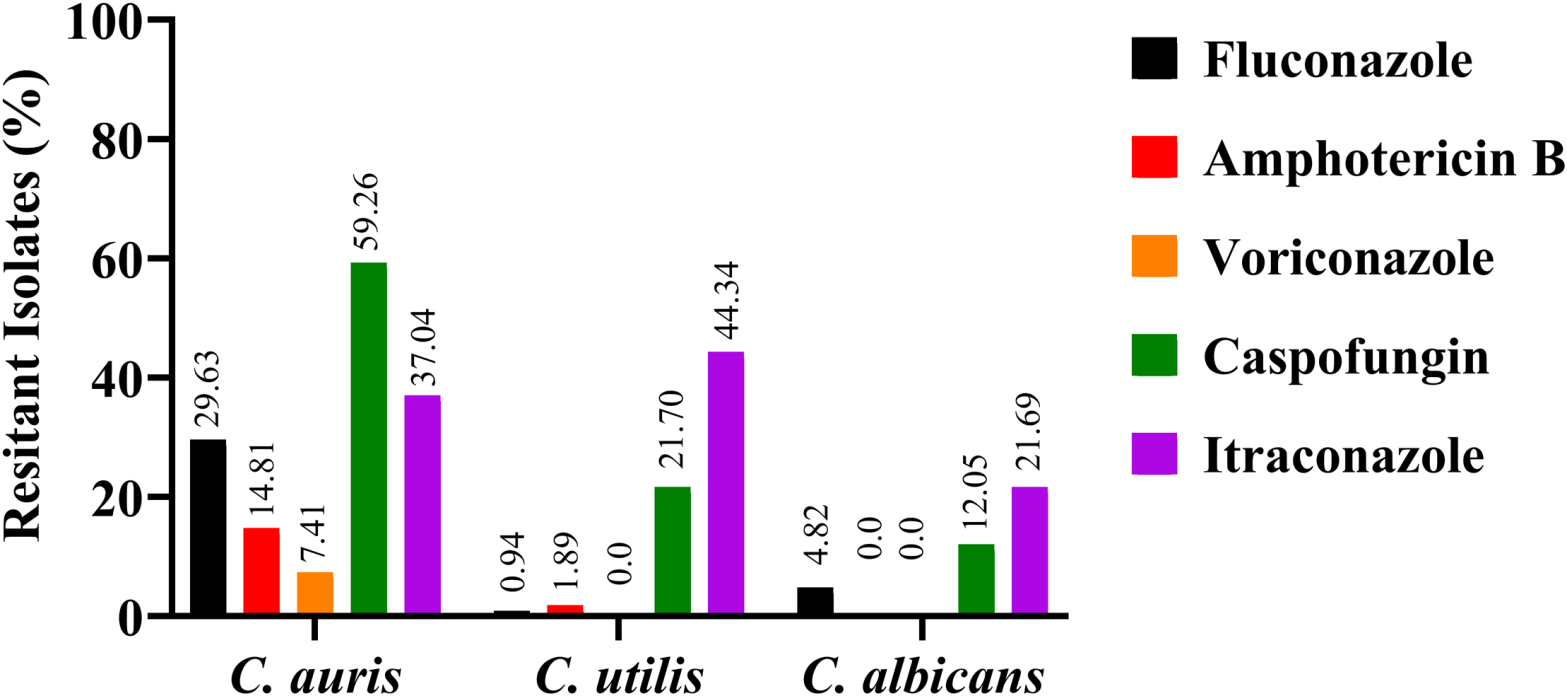
The bar diagram compares antifungal resistance of clinical isolates of *Candida auris*, *Candida utilis*, and *Candida albicans*, measured by the disc diffusion method. It shows the percentage of resistant isolates to fluconazole (black), amphotericin B (red), voriconazole (orange), caspofungin (green), and itraconazole (purple) for each species. *C. auris* had high multidrug resistance, especially to caspofungin (59.26%) and fluconazole (29.63%). *C. utilis* showed low resistance to fluconazole (0.94%), amphotericin B (1.89%), and voriconazole (0.0%), but higher resistance to caspofungin (21.70%) and itraconazole (44.34%). *C. albicans* showed the lowest resistance rates, with 4.82% for fluconazole, 0% for amphotericin B and voriconazole, 12.05% for caspofungin, and 21.69% for itraconazole. These findings highlight species differences, the threat of multidrug-resistant *C. auris*, and the clinical relevance of *C. utilis*.

The antifungal response across the three species is depicted in the comparative bar graph (**Figure 4**). Antifungal susceptibility profiling revealed distinct resistance patterns among the studied *Candida* species, highlighting essential implications for clinical treatment. For *Candida utilis* (n=106), resistance rates were notably low for fluconazole (0.94%) and amphotericin B (1.89%), with no isolates resistant to voriconazole. Moderate resistance to caspofungin (21.70%) was observed, while resistance to itraconazole was highest at 44.34%, making it the least effective against *C. utilis*. *Candida albicans* (n=83) showed slightly higher resistance to fluconazole (4.82%), no resistance to amphotericin B or voriconazole, and the lowest caspofungin resistance among the three species (12.05%). Itraconazole resistance (21.69%) was significantly lower than in *C. utilis*. This susceptibility profile confirms the clinical effectiveness of multiple antifungal agents against *C. albicans*.

In stark contrast, *Candida auris* (n=27) emerged as the most multidrug-resistant species. Resistance to fluconazole was high (29.63%), highlighting its limited effectiveness against this pathogen, and resistance to amphotericin B reached 14.81%. Although voriconazole resistance was relatively low (7.41%), caspofungin resistance was significantly higher (59.26%), the highest among all tested species. Itraconazole resistance (37.04%) was also considerable, making *C. auris* particularly alarming from a treatment perspective.

Overall, the findings highlight that *C. utilis* poses a challenge mainly with itraconazole, *C. albicans* remains broadly susceptible, and *C. auris* stands out as a significant multidrug-resistant threat, especially to fluconazole and caspofungin. This resistance landscape strongly emphasises the emerging challenge presented by *C. auris* and *C. utilis*, underscoring the importance of routine species-level diagnostics and targeted antifungal stewardship to optimise therapeutic efficacy and limit the spread of resistant *Candida* strains in hospital environments.

### 3.3 Impact of FeSO_4_ supplementation on antifungal susceptibility by broth microdilution

Broth microdilution assays were performed to assess the modulatory effect of ferrous sulphate (FeSO_4_) supplementation on antifungal susceptibility in clinical isolates of *C. albicans*, *C. auris*, and *C. utilis*. The findings demonstrated unique, species-specific, and drug-dependent responses to exogenous iron supplementation (**Table 1**).

**Table 1:** Species-specific and drug-specific modulation of antifungal susceptibility in *Candida* spp. under iron-rich conditions.

Candida spp.	Caspofungin	FeSO_4_ + Caspofungin	Fluconazole	FeSO_4_ + Fluconazole
*C. auris*	0.25	1.00	0.06^*^	0.06**^#^**
*C. utilis*	0.06	0.06	4	8
*C. albicans*	0.06	0.06	0.25	0.25

*Isovuconazole, ^#^FeSO4 160µg/mL + Isovuconazole. All drug concentrations are in µg/mL.

**Figure 5:**
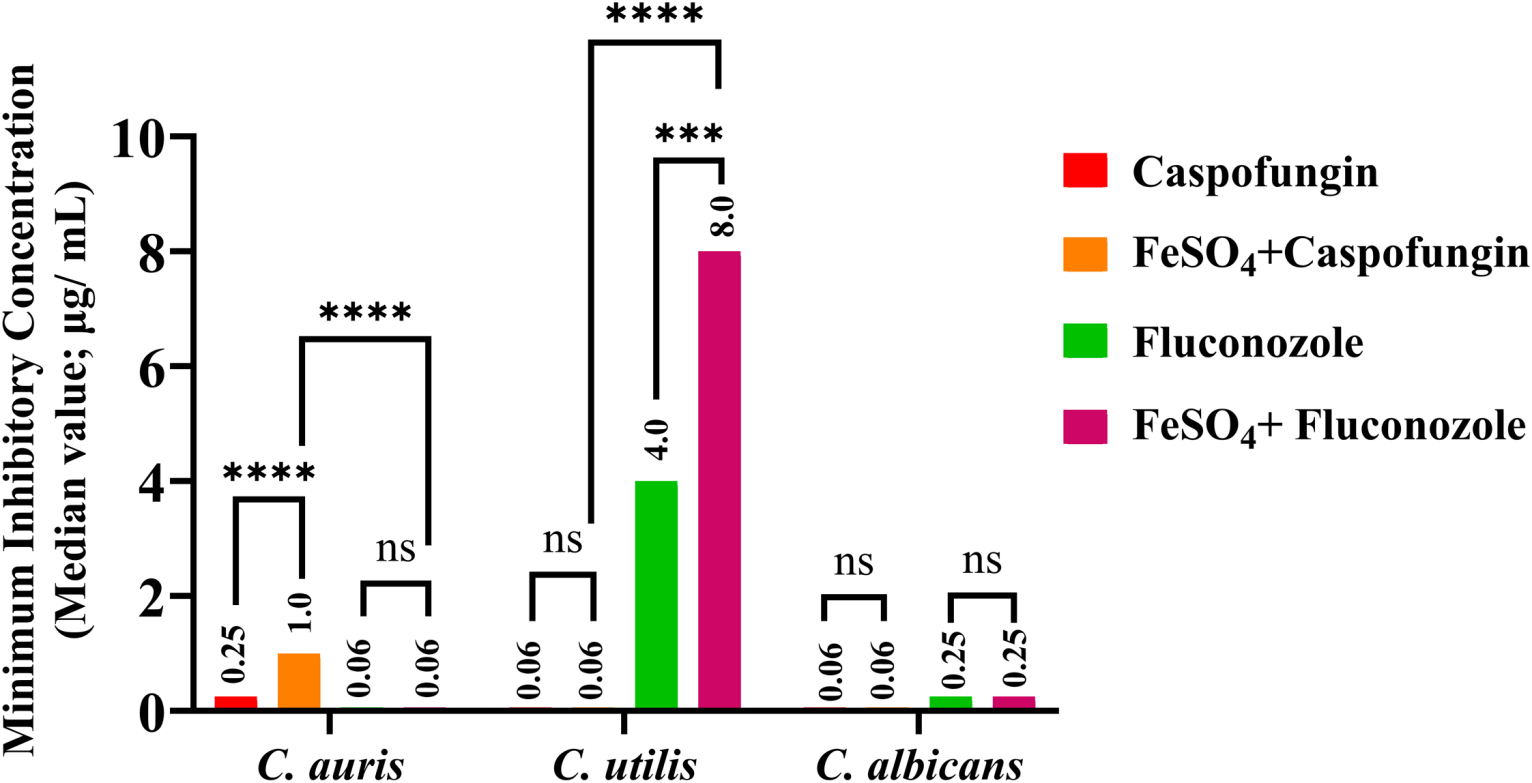
The figure shows how ferrous sulphate (FeSO_4_) affects MICs of caspofungin and fluconazole against *Candida* isolates. Median MICs (μg/mL) are depicted for caspofungin (red), fluconazole (green), FeSO_4_ + caspofungin (orange), and FeSO_4_ + fluconazole (magenta). Iron increases caspofungin MIC in *C. auris* and fluconazole MIC in C. utilis, with no significant change in *C. albicans*. Significance levels: ****p < 0.0001, ***p < 0.001, ns = not significant.

For *Candida auris*, baseline caspofungin MICs ranged from 0.0625 to 1 μg/mL. When supplemented with 160 μg/mL FeSO_4_, most isolates experienced a 1- to 2-fold increase in MICs, indicating a modest reduction in echinocandin susceptibility. Notably, one isolate showed a 16-fold increase in MIC under iron supplementation, suggesting a significant induction of caspofungin resistance in that strain. In contrast, isavuconazole MICs (0.03-2 μg/mL) remained unchanged across all isolates in iron-replete conditions, demonstrating that FeSO_4_ supplementation selectively impairs echinocandin activity without affecting triazole efficacy in *C. auris* (**Figure 5**).

In *C. utilis*, baseline fluconazole MICs ranged from 0.25 to 8 μg/mL. Upon supplementation with 160 μg/mL FeSO_4_, isolates with higher baseline MICs (2-8 μg/mL) exhibited significant 4- to 32-fold increases in fluconazole resistance, indicating a substantial iron-mediated boost in azole non-susceptibility. Conversely, caspofungin MICs (0.03-0.25 μg/mL) for *C. utilis* remained unaffected by iron supplementation, demonstrating that echinocandin susceptibility in this species is not modulated by iron availability (**Figure 5**).

Overall, these findings indicate that FeSO_4_ supplementation has an apparent, species-specific effect on antifungal susceptibility: *C. auris* develops increased resistance to caspofungin, while *C. utilis* shows heightened resistance to fluconazole. The lack of MIC changes for triazoles in *C. auris* and echinocandins in *C. utilis* emphasises distinct drug-specific pathways through which iron influences resistance phenotypes. These results highlight the importance of considering host iron status and environmental iron exposure in the clinical management of *Candida* infections, as iron supplementation may variably and significantly reduce antifungal effectiveness depending on the species and drug involved.

### 3.4 Virulence factor analysis: effect of FeSO_4_ supplementation

#### 3.4.1 Determination of phospholipase activity

**Figure 6:**
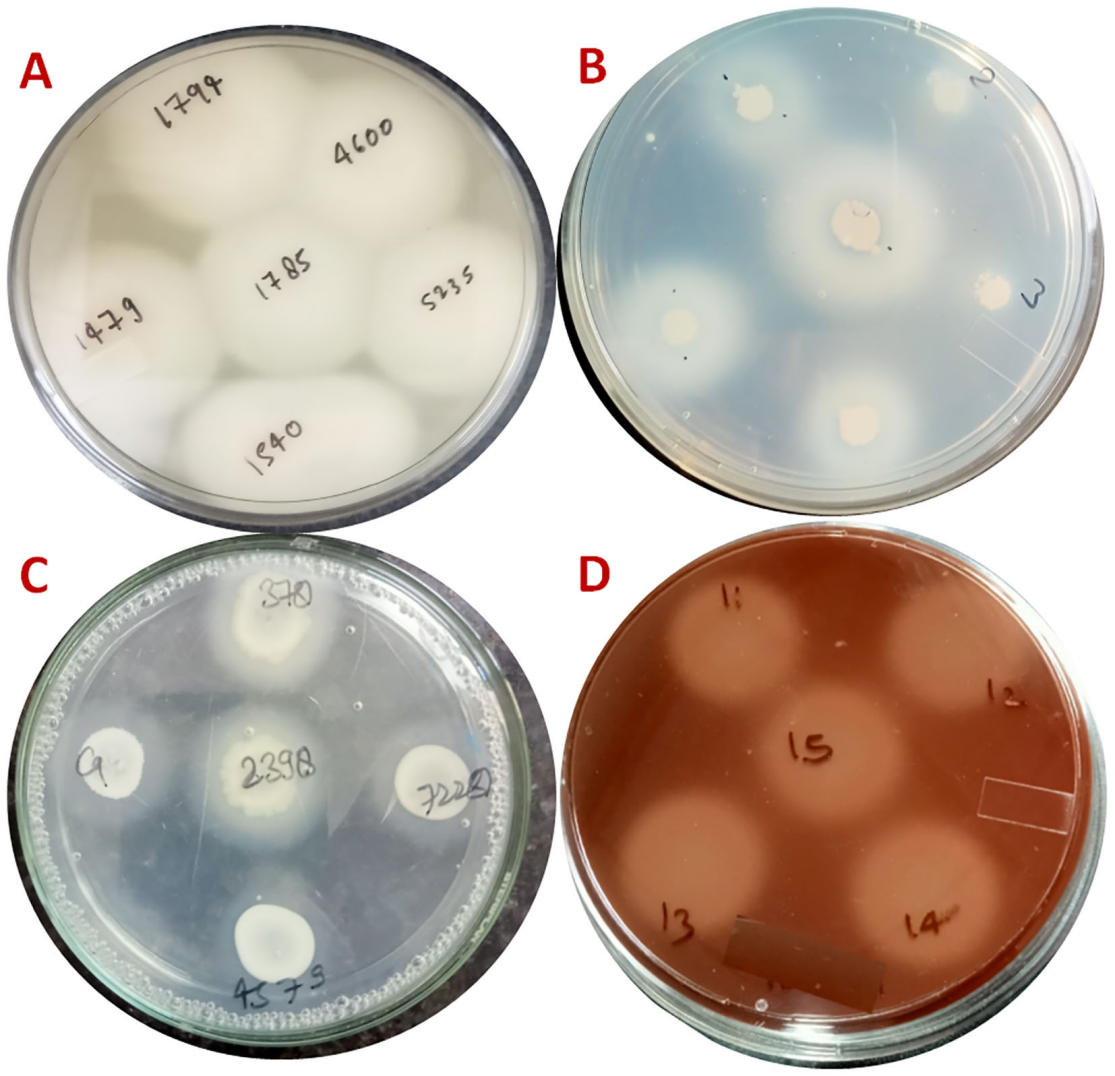
The image displays the effect of ferrous sulphate (FeSO_4_) supplementation on major extracellular virulence enzyme activities in clinical *Candida* isolates. **(A)** Phospholipase activity on egg yolk agar, showing precipitation zones around colonies; **(B)** Proteinase activity on albumin agar, evidenced by proteolytic clearance zones; **(C)** Esterase activity on Tween 80 opacity test medium, indicated by opaque precipitate halos; **(D)** Haemolysin activity on sheep blood agar, displayed as clear haemolytic zones around colonies. All assays were conducted both with and without FeSO_4_ at various concentrations. Quantification of activity was based on zone diameter measurements, with increased enzyme activity represented by larger precipitation or lytic zones.

Out of 106 *Candida utilis* samples, phospholipase activity was absent in all samples without iron sulphate. However, with 160 μg/mL iron sulphate, 54 samples showed vigorous positive phospholipase activity with precipitation zone (Pz) values ranging from 0.37 to 0.64. In comparison, 30 samples showed positive activity, with Pz values ranging from 0.65 to 0.8. Under these conditions, the minimum inhibitory concentration (MIC) of fluconazole increased by 1- to 6-fold, while the MIC of caspofungin remained unchanged regardless of iron sulphate presence. In 27 *Candida auris* isolates, phospholipase activity was consistently negative across all samples, both with and without iron sulphate at concentrations of 20-160 μg/mL (**Figure 6A**; **Table 2**).

**Table 2:** Influence of ferrous sulphate on various enzyme activities among *Candida* species.

Enzymatic activity	*C. auris*			*C. utilis*			*C. albicans*		
	SP %	P %	N %	SP %	P %	N %	SP %	P %	N %
Phospholipase									
Phospholipase+ FeSO_4_ (0µg/mL)	0.00	0.00	100.00	0.00	0.00	100.00	85.54	1.20	13.25
Phospholipase+ FeSO_4_ (80µg/mL)	0.00	0.00	100.00	0.00	0.00	100.00			
Phospholipase+ FeSO_4_ (160µg/mL)	0.00	0.00	100.00	48.11	31.13	20.75			
Proteinase									
Proteinase+ FeSO_4_ (0µg/mL)	96.29	00.00	3.71	0.00	0.00	0.00	89.15	6.024	4.81
Proteinase+ FeSO_4_ (80µg/mL)	100.00	00.0	00.00	100.00	0.00	0.00	100	00	00
Proteinase+ FeSO_4_ (160µg/mL)	100.00	00.00	00.00	100.00	0.00	0.00	100	00	00
Haemolysin									
Haemolysin+ FeSO_4_ (0µg/mL)	100,00	00.00	00.00	99.06	0.94	0.00	96.38	1.20	2.4
Haemolysin+ FeSO_4_ (80µg/mL)	100.00	00.00	00.00	100.00	0.00	0.00			
Haemolysin+ FeSO_4_ (160µg/mL)	100.00	00.00	00.00	100.00	0.00	0.00			
Esterase									
Esterase+ FeSO_4_ (0µg/mL)	77.77	18.51	3.703	0.00	0.00	100.00	90.36	3.6	6.02
Esterase+ FeSO_4_ (80µg/mL)	100.00	00.000	00.00	0.00	0.00	100.00			
Esterase+ FeSO_4_ (160µg/ml)	100.00	00,00	00.00	0.00	0.00	100.0			

Where SP stands for Strongly positive, P for Positive, and N for Negative.

#### 3.4.2 Determination of proteinase activity

Out of 106 *Candida utilis* samples, proteinase activity was absent without iron sulphate but became strongly positive with the addition of 80 μg/mL or 160 μg/mL FeSO_4_. The precipitation zone (Pz) values for proteinase activity ranged from 0.375 to 0.42 with 80 μg/mL FeSO_4_ and from 0.105 to 0.289 with 160 μg/mL FeSO_4_. Consistent with these results, the minimum inhibitory concentration (MIC) of fluconazole increased 1- to 6-fold in the presence of 160 μg/mL iron sulphate. In contrast, the MIC of caspofungin remained unchanged regardless of iron sulphate.

In contrast, for *Candida auris*, proteinase activity was strongly positive in 26 of 27 isolates without iron sulphate, with one sample showing no activity. However, all samples exhibited vigorous proteinase activity upon the addition of iron sulphate. The Pz values for *C. auris* ranged from 0.33 to 0.64 without iron sulphate, 0.30 to 0.50 with 80 μg/mL FeSO_4_, and 0.25 to 0.40 with 160 μg/mL FeSO_4_. Under these conditions, the MIC of caspofungin increased 1- to 4-fold with 160 μg/mL FeSO_4_, while the MIC of isavuconazole remained unaffected by the presence of iron sulphate.

For *Candida albicans*, proteinase activity was strongly positive in 74 of 83 samples without iron sulphate, with four samples showing no activity and 5 showing positive activity. However, all samples exhibited vigorous proteinase activity upon the addition of iron sulphate. The precipitation zone (Pz) values for *C. albicans* ranged from 0.40 to 0.64 without iron sulphate, 0.35 to 0.56 with 80 μg/mL FeSO_4_, and 0.23 to 0.45 with 160 μg/mL FeSO_4_ (**Figure 6B**; **Table 2**).

#### 3.4.3 Determination of esterase activity

In *Candida utilis*, esterase activity remained consistently negative, regardless of the presence or absence of 80 μg/mL or 160 μg/mL FeSO_4_. In *C. auris*, esterase activity was strongly positive in 21 isolates, with Pz values ranging from 0.48 to 0.64. In the presence of 80 and 160 μg/mL FeSO_4_, all 27 isolates exhibited strongly positive esterase activity, with Pz values ranging from 0.33 to 0.64 (**Figure 6C**; **Table 2**).

#### 3.4.4 Determination of haemolysin activity

In 106 *Candida utilis* isolates, haemolysin activity was strongly positive both with and without iron sulphate. The Pz values for haemolysin ranged from 0.285 to 0.75 without FeSO_4_, and with 80 μg/mL FeSO_4_, it ranged from 0.25 to 0.363, while with 160 μg/mL FeSO_4_, it ranged from 0.208 to 0.3. In *C. auris*, haemolysin activity was strongly positive in all samples, both with and without iron sulphate. The Pz values for haemolysin ranged from 0.27 to 0.50 without FeSO_4_ and with 80 μg/mL FeSO_4_, from 0.275 to 0.461 with 160 μg/mL FeSO_4_, and an additional reported range of 0.25 to 0.40 in the presence of FeSO_4_ (**Figure 6D**; **Table 2**).

### 3.5 Impact of ferrous sulphate on biofilm-forming capacity of *Candida* species

*C. utilis* exhibited robust biofilm formation at both control (0 µg/mL) and high (160 µg/mL) concentrations, with mean absorbance values of 1.013 and 1.025, respectively. The dataset across strains revealed no significant difference in biofilm quantification between untreated and treated groups (ANOVA: F = 0.011, p = 0.915), suggesting ferrous sulphate did not augment biofilm formation in *C. utilis*. Likewise, *C. auris*, a clinically relevant isolate, showed mean absorbance values of 0.799 (0 µg/ml) and 0.827 (160 µg/mL). ANOVA failed to demonstrate significant differences between groups (F = 0.044, p = 0.835), supporting the conclusion that ferrous sulphate does not meaningfully affect *C. auris* biofilm formation.

Notably, *C. albicans* displayed significantly lower biofilm formation relative to the other species, with mean absorbance values of 0.219 (0 µg/ml) and 0.206 (160 µg/ml). Statistical analysis (ANOVA: F = 1.03, p = 0.312) indicated no significant difference between the control and supplemented groups, suggesting minimal impact on biofilm formation.

An ANOVA-based analysis was performed to evaluate the biofilm-forming capacities of *C. utilis*, *C. auris*, and *C. albicans*. The findings demonstrated a markedly significant variation in the tendency for biofilm formation across these species in the presence of ferrous sulphate, supported by an ANOVA’s F-value of 1996.5 and a p-value of less than 0.0001. This finding aligns with the established comprehension of the distinct biofilm structures that define each species. Furthermore, the analysis revealed negligible variability within the experimental groups. The minimal variability observed within the group highlights the consistency and reproducibility of the biofilm quantification protocol utilised in this study, thereby affirming the reliability of the observed differences in biofilm formation across the tested *Candida* species.

Across all tested concentrations, ferrous sulphate did not significantly reduce biofilm formation in any of the *Candida* species analysed. However, *C. utilis* remains the most prolific biofilm producer, followed by *C. auris*, while *C. albicans* consistently formed substantially weaker biofilms. These results underscore pronounced interspecific variation in biofilm architecture and resilience and spotlight the recalcitrance of clinical yeast biofilms to exogenous chemical interventions and supplementation.

## 4 Discussion

The current study reveals that iron supplementation has variable effects on the expression of different virulence factors and on antifungal susceptibility among *Candida* species, particularly *Candida utilis*, *Candida auris*, and *Candida albicans*. Iron maintains robust biofilm biomass (no significant OD_570_ change, p>0.3) while selectively activating virulence enzymes (phospholipase, proteinase) in *C. utilis*. This aligns with other reports indicating that iron serves as an environmental signal that influences cell wall remodelling, enzyme secretion, and biofilm matrix structure. Elevated iron levels have been associated with decreased cell wall mannans and chitin, while increasing β-1,3-glucan, which promotes antifungal resistance and virulence (Comensoli et al., 2017; Tripathi et al., 2020). Conversely, *C. auris* exhibited strong biofilm formation and virulence enzyme production across a range of iron levels, illustrating its notorious adaptability and multidrug resistance.

Our study confirms that *C. utilis* forms robust biofilms in iron-rich environments, with iron supplementation preserving equivalent biomass (OD_570_1.013→1.025, p=0.915) while inducing phospholipase/proteinase activities. This pattern aligns with recent mechanistic studies of *C. albicans*, which show that high iron levels, in conjunction with carbon metabolism, facilitate hyphal growth, biofilm development, and cell wall modifications, thereby enhancing virulence (Tripathi et al., 2020). Similar findings have been observed in global surveys of hospital-acquired candidemia: biofilm-forming isolates, especially those in environments rich in iron or nutrients, present significant treatment challenges due to increased antifungal resistance (Amann et al., 2025).

Biofilm formation, a key feature of *Candida* pathogenicity, increases antifungal resistance and aids in evading immune responses (Kaur and Nobile, 2023). This study delineates the pivotal role of iron modulation in shaping biofilm−associated virulence, whereby iron−replete conditions preserve bulk biofilm biomass but remodel enzyme expression and antifungal susceptibility within these biofilms. Our findings demonstrate varied adaptations among species to iron-rich environments, emphasising the intricate pathobiology of these fungal pathogens and their relevance to clinical practice.

The robust biofilm formation exhibited by *C. utilis* under iron-replete conditions reinforces its recognition as a significant opportunistic pathogen possessing notable environmental resilience (Hazen et al., 1999; Ninan et al., 2021). Although *C. utilis* has traditionally been regarded for its industrial applications, the findings of this study establish its clinical relevance by demonstrating sustained robust biofilm biomass alongside iron-induced elevation of phospholipase/proteinase activities following ferrous sulphate supplementation (Buerth et al., 2016; Li et al., 2023). These results align with previous mechanistic research indicating that iron availability regulates cell wall remodelling and biofilm matrix reorganisation, processes associated with enhanced antifungal resistance and pathogenic potential. Notably, the strong correlation between iron-stimulated virulence enzyme production and a pronounced escalation in fluconazole minimum inhibitory concentration (up to 32-fold) underscores a direct mechanistic relationship between iron homeostasis and azole resistance, positioning iron as a critical environmental factor influencing therapeutic outcomes in candidemia.

Unlike other species, *C. auris* consistently formed strong biofilms and maintained high levels of virulence enzymes regardless of iron levels. This shows its natural resilience and ability to adapt to changing conditions within the host (Sanyaolu et al., 2022). Additionally, when exposed to exogenously supplemented iron, *C. auris* becomes even more resistant to caspofungin, reinforcing earlier findings that iron sensing influences key resistance mechanisms, such as efflux pump activity and cell wall stability, as reported for *C. albicans* (Simm et al., 2022; Pedras et al., 2025). The unchanged biofilm biomass and enzyme activity across different iron concentrations highlight *C. auris’s* potent pathogenic traits and help explain its ability to persist in hospitals despite aggressive antifungal treatments.

Notably, *C. albicans* displayed relatively lower biofilm densities and showed limited response to iron supplementation, indicating a stable yet potent virulence profile marked by consistently high proteinase and haemolysin activity. This observation is consistent with its recognised status as a predominant bloodstream pathogen possessing strong inherent pathogenic characteristics, while also suggesting that its virulence mechanisms may be less influenced by iron availability compared to non-albicans species (Ye et al., 2022).

**Figure 7:**
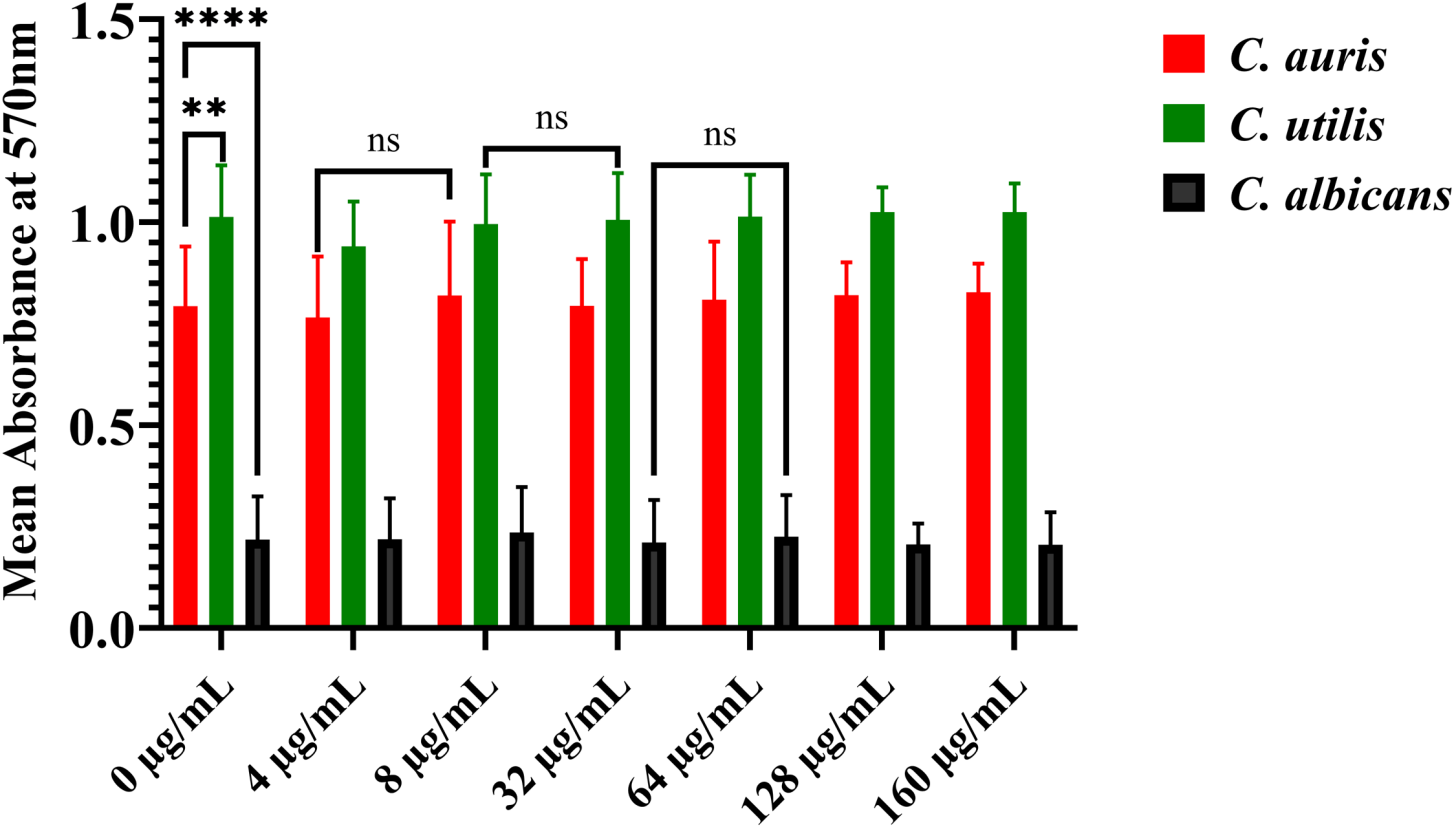
The bar diagram shows how ferrous sulphate (FeSO_4_) affects biofilm formation in *Candida auris* (red), *Candida utilis* (green), and *Candida albicans* (black), measured by crystal violet staining at 570 nm. Increasing FeSO_4_ (0-160 µg/mL) doesn’t significantly change biofilm formation in any species. However, differences are observed: *C. utilis* forms the most robust biofilm, *C. auris* is second, and *C. albicans* forms the weakest biofilm. Data are presented as mean absorbance ± SD; significance was assessed using ANOVA (**p < 0.01, ****p < 0.0001, ns = not significant).

Overall, the pronounced differences in how these species form biofilms and respond to iron highlight their unique evolutionary strategies tied to specific environments and host interactions. *C. utilis* demonstrates an iron-sensing virulence program that sustains prolific biofilm formation while upregulating invasive enzymes when iron is present- an adaptation that could be targeted by therapies like iron chelation or blocking iron uptake. In contrast, *C. auris* consistently forms robust biofilms and exhibits multidrug resistance, underscoring the pressing need for new antifungal drugs and rigorous infection control measures. Our results indicate *C. utilis* adapts to iron supplementation (160 μg/mL FeSO_4_) by maintaining high biofilm biomass while significantly activating phospholipases/proteinases. This adaptation directly contributes to higher fluconazole resistance, with MICs increasing 4- to 32-fold in iron-rich environments. No phospholipase activity was observed without iron, but over half of the *C. utilis* isolates showed vigorous activity of phospholipases, and all of the *C. utilis* isolates showed vigorous proteinase activity under high iron conditions, aligning with metabolic analyses that link high iron and carbon levels to increased pathogenicity in *Candida* species. These findings support previous research that environmental metal homeostasis influences phenotypic changes critical for infection and suggest potential for metabolic interventions, such as iron chelation or disruption, in anti-*Candida* treatment strategies (de Oliveira Santos et al., 2018; Simm et al., 2022). Notably, while FeSO_4_ did not significantly alter total biofilm biomass (crystal violet OD_570_; **Figure 7**), iron-replete conditions coincided with marked induction of biofilm-embedded virulence enzymes (e.g., C. utilis phospholipase: 0% → 48% strongly positive at 160 µg/mL FeSO_4_). This suggests iron promotes a qualitatively more virulent biofilm phenotype, maintaining structural integrity while enhancing tissue invasion capacity and antifungal tolerance, rather than quantitative biomass expansion. Such functional remodelling aligns with the *Candida* literature, in which iron modulates matrix composition and hyphal architecture without altering total adherent mass.

Compared to other species, *C. auris*, now recognised worldwide as a significant MDR fungus acquired in hospitals, consistently showed high levels of proteinase and haemolysin activity regardless of iron levels (Kim et al., 2024). When given additional iron, enzymatic activity and resistance to caspofungin further increased, with some isolates showing up to a 16-fold increase in MIC. These characteristics are similar to recent reports highlighting *C. auris’s* ability to adapt metabolically and withstand stress in challenging environments, including conditions of iron starvation or overload. This adaptability supports its ongoing colonization and spread in healthcare settings (Sabino et al., 2020). Our findings also align with reports showing that *C. auris* biofilms, which develop regardless of external iron levels, are highly resilient and resistant to various drugs.

*Candida albicans* remains the most prevalent pathogen in bloodstream infections globally, showing low initial resistance to azoles and polyenes, moderate resistance to echinocandins, and strong proteinase and haemolysin activity even without iron supplementation. The persistent activity of these key virulence enzymes contributes to tissue invasion, immune evasion, and the pathogen’s overall ability to cause severe infections. These enzymatic traits are intrinsic to *C. albicans* and play a critical role in its pathogenic potential (Mayer et al., 2013). Conversely, the *C. auris* isolates examined in our study exhibited notably high resistance rates: 29.6% to fluconazole and 59.3% to caspofungin. These findings are in agreement with contemporary global surveillance data, which consistently report elevated resistance levels in *C. auris* compared to other *Candida* species. The observed resistance patterns underscore the need for routine species-level diagnostics in clinical laboratories, as detailed identification is critical for informing effective antifungal therapy. Tailored treatment regimens must account for the fact that resistance in *C. auris* can be either an intrinsic characteristic of the species or induced by environmental factors, such as iron supplementation. This species- and environment-specific variability highlights the complexities involved in managing invasive candidiasis and emphasises the need for precision in both diagnosis and therapeutic strategy.

In contrast, resistance to echinocandins in *C. auris* increases with iron supplementation, while in *C. utilis*, resistance to azoles is preferentially enhanced in iron-rich conditions. Recent genomic and transcriptomic studies on *C. auris* have identified key resistance mechanisms, including point mutations in the *ERG11* and *FKS1* genes and the overexpression of multidrug efflux pumps. The increase in resistance associated with iron suggests that environmental factors, such as iron, may trigger upregulation and rewiring of biofilm−associated resistance networks, as supported by prior work linking iron availability to MAPK−mediated adhesion and cell wall remodelling, and to iron−dependent modulation of azole susceptibility and membrane properties in *Candida* species (Puri et al., 2014; Yang et al., 2024). Importantly, these results indicate a real risk that inadvertent iron overload in clinical settings (e.g., transfusions, underlying haemochromatosis) may fuel both *Candida* virulence and drug resistance, complicating therapeutic outcomes. The finding that caspofungin efficacy is reduced by iron overload in *C. auris* provides a mechanistic explanation for occasional antifungal failure in iron-rich clinical environments, a phenomenon recently demonstrated *in vitro* and discussed in current literature.

Phospholipase and proteinase activities were closely correlated with increased resistance to fluconazole, particularly in *C. utilis* isolates that were exposed to iron. These findings reinforce previous observations from both experimental and clinical studies, indicating that high phospholipase activity is often associated with antifungal resistance (Kumar et al., 2015; Zeitoun et al., 2024). This suggests that the overproduction of these enzymes may either drive or accompany mechanisms that confer drug tolerance, such as cell membrane remodelling and the activation of efflux pumps. This results in poorer patient outcomes and shorter survival times in infection models.

Our quantitative data show that resistance trajectories under iron supplementation are both species- and drug-specific: *C. auris* is particularly prone to caspofungin resistance upon iron exposure, while *C. utilis* demonstrates iron-driven amplification of fluconazole resistance. These findings are corroborated by other studies highlighting the mechanistic underpinnings that iron modulates membrane lipid composition, expression of multidrug transporters, and mitochondrial function, all of which collectively increase antifungal tolerance, such that perturbation of iron homeostasis and mitochondrial metabolism, not just nutrient availability, can be exploited therapeutically against MDR *Candida* (Tripathi et al., 2020; Simm et al., 2022). The observed resistance patterns are contextualised within broader epidemiology: *C. auris* resistance rates (fluconazole 30%, caspofungin 59%) align with Indian surveillance data (70-95% azole resistance, 10-40% echinocandin resistance; ICMR 2024) and exceed global averages (CDC: 90% fluconazole resistance, 5-20% caspofungin resistance), reflecting pressures from the South Asian clade (Indian Council of Medical Research, 2025; World Health Organization, 2025). *C. utilis* shows low fluconazole resistance (0.9%) but high itraconazole resistance (44%), consistent with European intensive care unit data (20-40% azole resistance). Iron increases azole minimum inhibitory concentrations (MICs) by 4-32-fold, a novel modulator not observed in *C. albicans* (fluconazole resistance 4.8%) (European Centre for Disease Prevention and Control & World Health Organization, 2025). These trends highlight the emergence of *C. utilis* and the clinical significance of iron, advocating for species-specific diagnostics and stewardship programs that account for iron levels.

The mechanistic underpinning of these results resonates with studies that describe how iron homeostasis sits at the fulcrum of both fungal metabolism and host-pathogen competition. Our data show that haemolysin activity in all *Candida* isolates is robust and largely unaffected by exogenous iron, suggesting that these pathogens are equipped to exploit host iron sources, a trait shown to be critical for virulence during deep-seated candidiasis (Srivastav et al., 2017). Furthermore, our finding that *C. auris* proteinase and esterase activities increase with iron supplementation, even in MDR clinical isolates, aligns with global analyses that pinpoint metabolic adaptability and secreted enzyme armamentarium as central determinants of invasive candidiasis severity. Although we did not perform *in vivo* infection models, the enzyme activities quantified here (phospholipases, proteinases, esterases, haemolysins) and biofilm formation are established virulence−associated traits in *Candida* spp., contributing to tissue invasion, immune evasion and poor therapeutic response in diverse infection models.

These multidimensional results provide evidence that iron availability is a central microenvironmental regulator of pathogenic traits, affecting biofilm−associated enzyme production and antifungal resistance, both of which are closely linked to pathogenicity in experimental and clinical studies. The integration of phenotypic and molecular analyses with the international literature corroborates the clinical significance and mechanistic implications of these findings, thereby directly informing therapeutic and diagnostic strategies for the management of complex *Candida* infections.

## 5 Conclusion

The findings of this study show that successful treatment of invasive candidiasis requires considering both the specific species causing the infection and the patient’s iron levels. It also highlights the importance of rapid molecular diagnostic tools and assessing environmental risks to help avoid treatment failure. Further research is warranted to elucidate the genomic and transcriptomic mechanisms underlying iron-mediated resistance in emerging *Candida* species. Integrating proteomic and metabolomic methodologies will enhance antifungal strategy development by enabling the identification of key metabolic vulnerabilities. The interaction among nutritional immunity, environmental exposure, and antifungal effectiveness is particularly significant for the development of adjunctive therapies, including chelation and metabolic inhibitors, as well as for advancing predictive diagnostic tools to support treatment stratification.

## Data Availability

The original contributions presented in the study are included in the article/supplementary material. Further inquiries can be directed to the corresponding authors.
